# Supplementary material for: Resolving the full spectrum of human genome variation using Linked-Reads
Source: Genome Res. 2019 Apr;29(4):635–45. doi: 10.1101/gr.234443.118 (PMC6442396; doi:10.1101/gr.234443.118)
Supplement: Supplemental Material [file supp_gr.234443.118_Supplemental_longranger-2.2.zip › longranger-2.2.tar.bz2!/lr2.2-public/lib/stan/cmdstan/stan/doc/api/html/rev_8hpp_source.html]

Stan: src/stan/agrad/rev.hpp Source File


|  |  |
| --- | --- |
|  | Stan  2.5.0  probability, sampling & optimization |


- Main Page
- Related Pages
- Namespaces
- Classes
- Files

- File List
- File Members

All Classes Namespaces Files Functions Variables Typedefs Enumerations Enumerator Friends Macros Pages

- src
- stan
- agrad

rev.hpp

Go to the documentation of this file.

1 #ifndef STAN\_\_AGRAD\_\_REV\_HPP

2 #define STAN\_\_AGRAD\_\_REV\_HPP

3

4 #include <stan/agrad/rev/var\_stack.hpp>

5 #include <stan/agrad/rev/chainable.hpp>

6 #include <stan/agrad/rev/vari.hpp>

7 #include <stan/agrad/rev/var.hpp>

8 #include <stan/agrad/rev/print\_stack.hpp>

9 #include <stan/agrad/rev/boost\_fpclassify.hpp>

10 #include <stan/agrad/rev/numeric\_limits.hpp>

11

12 #include <stan/agrad/rev/internal.hpp>

13 #include <stan/agrad/rev/operators.hpp>

14 #include <stan/agrad/rev/functions.hpp>

15 #include <stan/agrad/rev/error\_handling.hpp>

16 #include <stan/agrad/rev/ode.hpp>

17

18 #endif

operators.hpp

error\_handling.hpp

var\_stack.hpp

vari.hpp

boost\_fpclassify.hpp

ode.hpp

chainable.hpp

internal.hpp

print\_stack.hpp

var.hpp

functions.hpp

numeric\_limits.hpp


---

[ Stan Home Page ]

*© 2011–2014,
Stan Development Team.*
